# What limits a bystander’s action to perform cardiopulmonary resuscitation in China? Insights from a cross-sectional study conducted in Shaanxi Province

**DOI:** 10.3389/fpubh.2026.1902076

**Published:** 2026-08-27

**Authors:** Dongdong Li, Kun Wang, Qiang Fu, Yan Li, Wangang Guo, Xiaolan Guo, Xin Zhang, Yuchan Hu, Liping Liao, Zhuoni Ma, Xin Chen, Miaomiao Cheng

**Affiliations:** 1 Department of Cardiology, Tangdu Hospital of Fourth Military Medical University, Xi’an, Shaanxi, China; 2 Department of Cardiology, Weinan Second Hospital, Weinan, Shaanxi, China; 3 Department of Special Medical Service, Army Medical Center of Army Medical University, Chongqing, China; 4 Department of Nursing, Xi’an Gem Flower Changqing Staff Hospital, Xi’an, Shaanxi, China

**Keywords:** barriers to action, cardiopulmonary resuscitation, family caregivers, health knowledge, attitudes, practice, out-of-hospital cardiac arrest

## Abstract

**Background:**

Rate of bystander-initiated cardiopulmonary resuscitation (CPR) remains critically low in China. Identifying the underlying reasons may help reduce mortality from out-of-hospital cardiac arrest (OHCA).

**Methods:**

A cross-sectional study was conducted among 572 participants in Shaanxi, China. A researcher-developed questionnaire was used to assess four domains: ability to recognize the need for CPR, willingness to perform CPR, internal concerns about initiating CPR, and actual CPR competence. Subgroup analyses were performed across demographic strata. Willingness and concerns regarding CPR for relatives versus strangers were distinguished and compared.

**Results:**

Of the participants, 73.95% demonstrated the ability to recognize the need to perform CPR. Subgroup analysis revealed individuals aged ≥ 50 years, rural residents, and those with lower educational attainment had relatively poorer ability. Regarding willingness to perform CPR, over 94% of participants reported willingness to act for both relatives and strangers. However, the intensity was significantly greater for relatives than for strangers (8.84 ± 2.35 *vs.* 7.43 ± 2.53, *p* < 0.001). Notably, individuals with lower educational levels exhibited weaker willingness for both relatives and strangers. A high proportion of participants reported internal concerns about initiating CPR (91.78% for relatives, 93.88% for strangers), which was especially pronounced among spouses of high-risk patients. Lack of confidence in skills was the most frequently cited barrier. Additionally, participants reported more concerns regarding psychological fear, poor physical condition, medical litigation, and sanitary issues when the victim was a stranger. Finally, rural residents and those with primary education or below demonstrated poorer CPR knowledge scores.

**Conclusion:**

Overall, the following issues should be prioritized. First, lack of confidence in skills—which may stem from poor actual competence—is widely cited. Second, individuals have greater concerns regarding psychological fear, poor physical condition, medical litigation, and sanitary issues when the victim is a stranger. Finally, special attention should be paid to populations characterized by age ≥ 50 years, lower educational level, and rural residence, as they demonstrated a tendency toward poorer performance across all assessed domains.

## Introduction

Rate of bystander-initiated cardiopulmonary resuscitation (CPR) in China remains critically low. The BASIC-OHCA registry reported a national rate of 20.3%, while some regional studies have documented rates as low as 4.8% ~ 17.0% (1–3). These figures lag substantially behind the rates of 39.6% ~ 58.5% reported in developed countries (4, 5). Several factors hindering bystander action have been identified, including lack of knowledge of CPR procedures, concerns about legal liability, fear of contagious diseases, fear of harming the victim, psychological discomfort, and others (6–20). However, previous studies have typically examined individual factors in isolation, lacking comprehensive and holistic analysis. Consequently, it remains unclear which of these factors constitute the primary barriers. Clarifying this issue is essential for designing targeted interventions to improve bystander CPR rates and reduce mortality from out-of-hospital cardiac arrest (OHCA). To guide this investigation, we adopted a simplified “three-step” framework—awareness, decision, and action—synthesized from the existing theoretical models (21–24). With this framework, we systematically and comprehensively assessed participants’ ability to recognize the need for CPR, their willingness to act, internal concerns, and actual CPR competence in this study. In addition, we distinguished and compared willingness and internal concerns regarding performing CPR on relatives versus strangers.

## Methods

### Study design

This was a multi-center, cross-sectional study conducted between July and October 2024 across three hospitals in two cities (Xi’an and Weinan) of Shaanxi Province in China: Tangdu Hospital, Weinan Second Hospital, and Xi’an Gem Flower Changqing Staff Hospital. All centers followed standardized protocols for participant enrollment and data collection.

### Participant enrollment

Given the need to investigate the subjects’ attitudes about CPR on their relatives, we selected relatives of patients at high-risk for OHCA as the study subjects. All the patients were consecutively screened from the inpatients of cardiovascular departments diagnosed with at least one of the following disease: heart failure, coronary artery disease, congenital structural heart disease, hypertrophic cardiomyopathy, dilated cardiomyopathy, atrial fibrillation, long QT syndrome, Brugada syndrome, frequent premature ventricular contractions, ventricular tachycardia, *etc.* (25–28). Subsequently, their family members who were accompanying them during hospitalization were invited to participate in this study. Eligibility was determined based on the following criteria.

Inclusion criteria: (a) being a close family member of the patient, categorized as spouse, offspring, or others (including parents, siblings, and other extended relatives); (b) cohabiting with the patient for at least 50% of one year; (c) Aged 18 to 75 years; (d) Provision of written informed consent.

Exclusion criteria: (a) Diagnosis of a mental or intellectual disability; (b) Completion of the survey with > 20% missing data; (c) Provision of responses deemed inconsistent or implausible after verification by two independent investigators. Notably, the followings could be deemed as inconsistent or implausible: the answer choices are all A/B/C/D or follow a regular sequence; cases include those who answered “No” to whether they have heard of CPR yet answered “Yes” to whether they have received training or performed CPR; those who answered “No” to whether they have received training yet indicated a number of training sessions other than zero.

### Sample size calculation

Sample size estimation was calculated with the formula:


$$n=\frac{Z^{2}P(1-P)}{d^{2}}$$

We adopted the most conservative estimate with *p* = 0.5, Z = 1.96, and d = 0.05. This yielded a minimum sample size of 385 participants.

### Data collection and outcome measures

Data were collected using a researcher-developed questionnaire assessing five key domains: (a) Baseline characteristics, including gender, age, urban/rural residence, education level (categorized as primary or below, secondary, or college or above), kinship to the patient (spouse, offspring or others), and cohabitation duration; (b) ability to recognize the need for CPR—whether the participant had heard of CPR and could recognize the need to perform it when sudden cardiac arrest (CA) occurred. It was assessed by two questions detailed in Supplementary material, only those both answered correctly were considered to have the ability to recognize; (c) CPR willingness intensity, measured on a self-reported 0 ~ 10 scale, with scores of 8 ~ 10, 4 ~ 7, and 0 ~ 3 classified as strong, moderate, and weak willingness, respectively; (d) Internal concerns, encompassing lack of confidence in skills, physical limitations, concerns about medical liability, sanitation concerns, and psychological fear—which were screened out from various factors in the previous pilot study; (e) CPR competency-related information included prior CA witness experience, previous CPR performance on a human, CPR training history (recency and frequency), automated external defibrillator (AED) recognition, and score of a validated 20-item quick knowledge test (Supplementary material). Notably, the domains of (b) to (e) were based on the simplified “three-step” framework. (b) reflects the awareness step, (c) and (d) reflects the decision step, and (e) reflects the action step.

The above information was compared across different demographic strata. Attitudes toward relatives and strangers were compared.

### Reliability and validity tests

The following items are based on objective facts: gender, age, residence, kinship, educational level, cohabitation duration with the patient, history of witnessing CA, history of performing CPR on a person, training experience, time since last training, number of training sessions, and awareness of AED. These items were reviewed by our expert panel and deemed clear and unambiguous. Therefore, reliability and validity testing about these items was not performed.

Test–retest reliability for the willing intensity, internal concerns and 20-item quick knowledge test was assessed. The intraclass correlation coefficients for willing intensity and 20-item quick knowledge test were 0.885 (95% CI: 0.811–0.930, *p* < 0.001) and 0.908 (95% CI: 0.817–0.955, *p* < 0.001), while *κ* for internal concerns was 0.683 (*p* < 0.001). These results indicated excellent temporal stability.

Content validity for the willing intensity, internal concerns and 20-item quick knowledge test was also assessed. For the willing intensity, the item-level content validity index (I-CVI) ranged from 0.80 to 1.00, while the scale-level content validity index based on average (S-CVI/Ave) was 0.90. For the internal concerns, the I-CVI ranged from 0.80 to 1.00, while the S-CVI/Ave was 0.96. For the 20-item CPR knowledge test, the I-CVI ranged from 0.80 to 1.00, while the S-CVI/Ave was 0.96.

### Statistical analysis

Continuous data are expressed as mean ± standard deviation, and categorical data as number (percentage). Normality and homogeneity of variance were assessed to guide the choice of statistical tests. For binary paired comparisons (McNemar test), we calculated the effect size Cohen’s g. Effect sizes were interpreted as: 0.1 = small, 0.3 = medium, and 0.5 = large. For paired categorical data with more than two categories (McNemar-Bowker test), we calculated effect size Cohen’s *κ*. Values of 0.00–0.20 indicated slight agreement, 0.21–0.40 fair, 0.41–0.60 moderate, 0.61–0.80 substantial, and 0.81–1.00 almost perfect agreement. For paired nonparametric comparisons (Wilcoxon signed-rank tests), we calculated the effect size r. r = 0.1, 0.3, and 0.5 were considered small, medium, and large effects, respectively. Linear regression or binary logistic regression was used to obtain *β* coefficients or odds ratios (OR) along with their 95% confidential intervals (CI) in univariate or multivariate regression analyses. To address multiple comparisons, we applied the Benjamini-Hochberg procedure to control the false discovery rate (FDR) at 5%. All analyses were performed using SPSS version 26.0 (*IBM Corp.*). Statistical significance was set at a two-sided *p* < 0.05 for the main analyses, and at an FDR-adjusted *p* < 0.05 for the multiple comparisons.

## Results

### Baseline characteristics

A total of 855 questionnaires were distributed, 840 were returned (response rate 98.25%). After applying the inclusion and exclusion criteria, 572 participants were enrolled. 572 participants were finally enrolled. All questionnaires were fully completed, without any missing data. The cohort included 219 males (38.29%) with a mean age of 50.96 ± 12.19 years. 398 (69.58%) resided in urban areas. Regarding kinship, the majority were spouses (352, 61.54%), followed by offspring (197, 34.44%). Parents (11, 1.92%) and other relatives (12, 2.10%) represented a small proportion. Educational attainment was predominantly at the secondary level (382, 66.78%), followed by college level or above (125, 21.85%) and primary level or below (65, 11.36%). Participants reported spending 93.23% ± 12.42% of their time with the OHCA high-risk patient (Table 1).

**Table 1:** Baseline characteristics of the participants.

Items	*N* = 572
Male, *n* (%)	219 (38.29%)
Mean age, (years, mean ± SD)	50.96 ± 12.19
Age, *n* (%)	
< 50	245 (42.83%)
≥ 50	327 (57.17%)
Urban area, *n* (%)	398 (69.58%)
Kinship with the high-risk OHCA patient, *n* (%)	
Spouse	352 (61.54%)
Offspring	197 (34.44%)
Others ^a^	23(4.02%)
Educational level, *n* (%)	
Primary or lower	65 (11.36%)
Secondary	382 (66.78%)
College or higher	125 (21.85%)
Mean cohabitation duration with the high-risk OHCA patient, (%, mean ± SD)	93.23 ± 12.42
Cohabitation duration with the high-risk OHCA patient, *n* (%)	
< 100%	172 (30.07%)
100%	400 (69.97%)

^a^Including parents, siblings, and other extended relatives. OHCA, out-of-hospital cardiac arrest. SD, standard deviation.

### Ability to recognize the need to perform CPR

423 participants (73.95%) demonstrated the ability to recognize the need to perform CPR for OHCA. Subgroup analysis revealed significantly poorer ability among individuals ≥ 50 years, rural residents, those with lower educational attainment and spousal kinship compared to their counterparts. After adjusted for factors of gender, age, residence, education level, and kinship, individuals ≥ 50 years, rural residents, and those with lower educational attainment still showed a poorer ability than their counterparts (< 50 *vs.* ≥ 50: aOR = 0.556, 95% CI = 0.331–0.935; rural *vs.* urban: aOR = 2.012, 95% CI = 1.309–3.092; primary or below *vs.* secondary: aOR = 2.224, 95% CI = 1.256–3.939; primary or below *vs.* college or above: aOR = 3.261, 95% CI = 1.436–7.405) (Table 2).

**Table 2:** Comparisons of ability to recognize the need for CPR across subgroups by univariate and multivariate models adjusted for gender, age, residence, education level, and kinship.

Items	n/N (%)	Crude OR (95% CI), *p*, *P*_FDR_	Adjusted OR (95% CI) ^a^, *p*, *P*_FDR_
Overall	423/572 (73.95%)	—	—
Gender			
Male	161/219 (73.52%)	Ref	Ref
Female	262/353 (74.22%)	1.037 (0.707, 1.522), 0.852, 0.852	1.066 (0.709, 1.604), 0.757, 0.958
Age			
< 50	206/245 (84.08%)	Ref	Ref
≥ 50	217/327 (66.36%)	0.373 (0.247, 0.564), < 0.001, < 0.001	0.556 (0.331, 0.935), 0.027, 0.047
Residence			
Rural areas	102/174 (58.62%)	Ref	Ref
Urban areas	321/398 (80.65%)	2.943 (1.990, 4.351), < 0.001, < 0.001	2.012 (1.309, 3.092), 0.001, 0.007
Education level			
Primary or below	31/65 (47.69%)	Ref	Ref
Secondary	283/382 (74.08%)	3.135 (1.831, 5.368), < 0.001, < 0.001	2.224 (1.256, 3.939), 0.006, 0.014
College or above	109/125 (87.20%)	7.472 (3.653, 15.284), < 0.001, < 0.001	3.261 (1.436, 7.405), 0.005, 0.014
Kinship			
Spouse	248/352 (70.45%)	Ref	Ref
Offspring	157/197 (79.70%)	1.646 (1.086, 2.495), 0.019, 0.022	1.036 (0.630, 1.706), 0.888, 0.958
Others	18/23 (78.26%)	1.510 (0.546, 4.174), 0.019. 0.022	1.030 (0.343, 3.095), 0.958, 0.958

^a^Adjusted for gender, age, residence, education level, and kinship to the high-risk patient. CPR, cardiopulmonary resuscitation. OR, odds ratio. CI, confidential interval. FDR, false discovery rate.

We then examined the interaction between education and residence, education and age, residence and age. The interaction term was not statistically significant (P for interaction = 0.788, 0.476, 0.329, respectively), suggesting that education, residence and age independently, rather than synergistically, influenced this ability.

### Willingness to perform CPR

A total of 549 participants (95.63%) reported willingness to perform CPR for relatives, and 542 (94.76%) for strangers, with no significant difference in the binary willingness measure (*p* = 0.180). However, when analyzing the intensity of willingness, 509 participants (88.99%) reported strong willingness to perform CPR for relatives, compared with 343 (59.97%) for strangers. The difference was statistically significant (*p* < 0.001). Quantitative analysis yielded consistent findings. The mean willing intensity for relatives (8.84 ± 2.35) fell within the “strong” range, whereas that for strangers (7.43 ± 2.53) was within the “moderate” range. The difference was also statistically significant (*p* < 0.001) (Table 3).

**Table 3:** Participants’ willingness to perform CPR for relatives versus strangers by qualitative and quantitative analyses.

Items	Reported willingness to perform CPR^a^	Qualitative analysis of willing intensity^b^			Quantitative analysis of willing intensity^c^
		Strong (8 ~ 10)	Moderate (4 ~ 7)	Weak (0 ~ 3)	
For relatives, *n* (%)	547 (95.63%)	509 (88.99%)	32 (5.59%)	31 (5.42%)	8.84 ± 2.34
For strangers, *n* (%)	542 (94.76%)	343 (59.97%)	192 (33.57%)	37 (6.47%)	7.43 ± 2.53
Cohen’s g/Cohen’s κ/r	0.278	0.295			0.568
*p*	0.180	< 0.001			< 0.001

^a^Analyzed by McNemar test. ^b^Analyzed by McNemar Bowker test. ^c^Analyzed by Wilcoxon Signed-Rank Test. CPR, cardiopulmonary resuscitation.

The study also indicated that individuals with primary educational level or below showed weaker willingness than those with college education level or above for both relatives (primary or below *vs.* college or above: adjusted *β* = 1.551, 95% CI = 0.749–2.353) and strangers (primary or below *vs.* college or above: adjusted *β* = 1.790, 95% CI = 0.927–2.654) after adjusted for factors of gender, age, residence, education level, and kinship (Table 4).

**Table 4:** Comparisons of willingness to perform CPR across subgroups by univariate and multivariate models adjusted for gender, age, residence, education level, and kinship.

Items	For relatives			For strangers		
	Willing intensity (mean ± SD)	Crude β (95%CI), *p*, *P*_FDR_	Adjusted *β* (95%CI)^a^, *p*, *P*_FDR_	Willing intensity (mean ± SD)	Crude β (95%CI), *p*, *P*_FDR_	Adjusted *β* (95%CI)^a^, *p*, *P*_FDR_
Gender						
Male	8.87 ± 2.33	Ref	Ref	7.44 ± 2.56	Ref	Ref
Female	8.82 ± 2.36	−0.046 (−0.443, 0.350), 0.820, 0.820	0.016 (−0.380, 0.413), 0.936, 0.936	7.42 ± 2.52	−0.016 (−0.444, 0.412), 0.941, 0.941	0.040, (−0.387, 0.466), 0.855, 0.855
Age						
< 50	9.08 ± 2.17	Ref	Ref	7.50 ± 2.53	Ref	Ref
≥ 50	8.66 ± 2.46	−0.417 (−0.805, −0.029), 0.035, 0.082	−0.076 (−0.563, 0.410), 0.758, 0.884	7.37 ± 2.53	−0.129 (−0.549, 0.291), 0.547, 0.760	0.415, (−0.108, 0.939), 0.120, 0.280
Residence						
Rural areas	8.64 ± 2.46	Ref	Ref	7.16 ± 2.60	Ref	Ref
Urban areas	8.93 ± 2.29	0.281 (−0.137, 0.699), 0.188, 0.263	−0.106 (−0.558, 0.347), 0.647, 0.884	7.55 ± 2.52	0.384 (−0.067, 0.836), 0.095, 0.222	0.047, (−0.441, 0.534), 0.850, 0.855
Education level						
Primary or below	7.89 ± 3.20	Ref	Ref	6.69 ± 3.05	Ref	Ref
Secondary	8.80 ± 2.35	0.911 (0.303, 1.520), 0.003, 0.011	0.912 (0.270, 1.555), 0.005, 0.018	7.28 ± 2.48	0.588 (−0.069, 1.244), 0.079, 0.222	0.662 (−0.029, 1.354), 0.061, 0.214
College or above	9.44 ± 1.52	1.548 (0.854, 2.241), < 0.001,0.007	1.551 (0.749, 2.353), < 0.001, 0.007	8.26 ± 2.17	1.572 (0.824, 2.320), < 0.001, 0.007	1.790 (0.927, 2.654), < 0.001, 0.007
Kinship						
Spouse	8.74 ± 2.42	Ref	Ref	7.38 ± 2.53	Ref	Ref
Offspring	9.04 ± 2.14	0.291 (−0.118, 0.701), 0.163, 0.263	0.084 (−0.384, 0.552), 0.724, 0.884	7.48 ± 2.54	0.102 (−0.341, 0.545), 0.651, 0.760	0.091 (−0.413, 0.595), 0.723, 0.855
Others	8.84 ± 2.34	−0.136 (−1.127, 0.855), 0.788, 0.820	−0.362 (−1.371, 0.646), 0.481, 0.884	7.43 ± 2.53	0.451 (−0.620, 1.522), 0.409, 0.716	0.339 (−0.747, 1.426), 0.540, 0.855

^a^Adjusted for gender, age, residence, education level, and kinship to the high-risk patient. CPR, cardiopulmonary resuscitation. SD, standard deviation. CI, confidential interval. FDR, false discovery rate.

### Internal concerns about initiating CPR

525 (91.78%) reported internal concerns about initiating CPR for relatives, while 537 (93.88%) for strangers. The difference was statistically significant (*p* = 0.036). Five reasons for internal concerns were then evaluated. For relatives, lack of confidence in skills was the most reported (488, 85.31%), followed by psychological fear (163, 28.50%), poor physical condition (26, 4.55%), medical litigation concerns (14, 2.45%) and sanitary concerns (4, 0.70%). For strangers, lack of confidence in skills was also the most reported (498, 87.06%), followed by psychological fear (269, 47.03%), medical litigation concerns (147, 25.70%), poor physical condition (40, 6.99%) and sanitary concerns (20, 3.50%). Lack of confidence in skills was reported far more frequently than other reasons for both relatives and strangers (*p* < 0.001 for all). It showed that the internal concerns were reported more frequently for strangers in aspects of psychological fear, poor physical condition, medical litigation concerns and sanitary concerns than for relatives (47.03% *vs.* 28.50%, *p* < 0.001; 6.99% *vs.* 4.55%, *p =* 0.013; 25.70% *vs.* 2.45%, *p* < 0.001; 3.50% *vs.* 0.70%, *p* < 0.001; respectively), whereas there was no significant difference for lack of confidence in skills (87.06% *vs.* 85.31%, *p =* 0.154) (Table 5).

**Table 5:** Internal concerns about initiating CPR for relatives versus strangers, and comparison across different reasons.

Items	For relatives		For strangers		Comparison between for relatives and strangers, OR (95%CI)^a^, *p*
	*n* (%)	Comparison among reasons, OR (95%CI), *p*	*n* (%)	Comparison among reasons, OR (95%CI), *p*	
Reported internal concerns	525 (91.78%)	—	537 (93.88%)	—	2.500 (1.101, 5.678), 0.036
Reasons:					
Lack of confidence in CPR skills	488 (85.31%)	Ref	498 (87.06%)	Ref	1.667 (0.879, 3.162), 0.154
Psychological fear	163 (28.50%)	0.087 (0.060, 0.126), < 0.001	269 (47.03%)	0.095 (0.062, 0.144), < 0.001	5.240 (3.416, 8.037), < 0.001
Poor physical condition	26 (4.55%)	0.011 (0.004, 0.026), < 0.001	40 (6.99%)	0.009 (0.003, 0.023), < 0.001	3.000 (1.275, 7.057), 0.013
Medical litigation concerns	14 (2.45%)	0.006 (0.002, 0.020), < 0.001	147 (25.70%)	0.054 (0.034, 0.085), < 0.001	134.000 (18.738, 958.280), < 0.001
Sanitary concerns	4 (0.70%)	0.004 (0.001, 0.017), < 0.001	20 (3.50%)	0.010 (0.004, 0.025), < 0.001	17.000 (2.262, 127.745), < 0.001

^a^For relatives was chosen as the reference. McNemar test for all analyses. CPR, cardiopulmonary resuscitation. OR, odds ratio. CI, confidential interval.

Subgroup analysis revealed that spousal kinship of the high-risk patients reported much higher internal concerns than offspring for both relatives (spouse *vs.* offspring: aOR = 0.308, 95% CI = 0.150–0.629) and strangers (spouse *vs.* offspring: aOR = 0.221, 95% CI = 0.098–0.500) after adjusted for factors of gender, age, residence, education level, and kinship (Table 6).

**Table 6:** Comparisons of internal concerns about initiating CPR across subgroups by univariate and multivariate models adjusted for gender, age, residence, education level, and kinship.

Items	For relatives			For strangers		
	n/N (%)	Crude OR (95%CI), *p*, *P*_FDR_	Adjusted OR (95%CI)^a^, *p*, *P*_FDR_	n/N (%)	Crude OR (95%CI), *p*, *P*_FDR_	Adjusted OR (95%CI)^a^, *p*, *P*_FDR_
Gender						
Male	202/219 (92.24%)	Ref	Ref	205/219 (93.61%)	Ref	Ref
Female	323/353 (91.50%)	0.906 (0.487, 1.685), 0.755, 0.755	0.765 (0.404, 1.446), 0.410, 0.478	332/353 (94.05%)	1.080 (0.537, 2.171), 0.830, 0.968	0.935 (0.457, 1.912), 0.853, 0.895
Age						
< 50	226/245 (92.24%)	Ref	Ref	233/245 (95.10%)	Ref	Ref
≥ 50	299/327 (91.44%)	0.898 (0.489, 1.648), 0.728, 0.755	0.434 (0.195, 0.965), 0.041, 0.105	304/327 (92.97%)	0.681 (0.332, 1.396), 0.294, 0.655	0.311 (0.126, 0.769), 0.011, 0.039
Residence						
Rural areas	155/174 (89.08%)	Ref	Ref	161/174 (92.53%)	Ref	Ref
Urban areas	370/398 (92.96%)	1.620 (0.878, 2.987), 0.122, 0.427	2.035 (1.017, 4.073), 0.045, 0.105	376/398 (94.47%)	1.380 (0.678, 2.807), 0.374, 0.655	1.280 (0.582, 2.815), 0.539, 0.895
Education level						
Primary or below	61/65 (93.85%)	Ref	Ref	61/65 (93.85%)	Ref	Ref
Secondary	352/382 (92.15%)	0.769 (0.262, 2.261), 0.634, 0.755	0.617 (0.201, 1.895), 0.399, 0.478	358/382 (93.72%)	0.978 (0.328, 2.917), 0.968, 0.968	0.925 (0.291, 2.936), 0.895, 0.895
College or above	112/125 (89.60%)	0.565 (0.177, 1.808), 0.336, 0.755	0.291 (0.075, 1.125), 0.074, 0.130	118/125 (94.40%)	1.105 (0.311, 3.923), 0.877, 0.968	0.749 (0.171, 3.275), 0.701, 0.895
Kinship						
Spouse	331/352 (94.03%)	Ref	Ref	338/352 (96.02%)	Ref	Ref
Offspring	172/197 (87.31%)	0.436 (0.237, 0.802), 0.008, 0.056	0.308 (0.150, 0.629), 0.001, 0.007	178/197 (90.35%)	0.388 (0.190, 0.792), 0.009, 0.063	0.221 (0.098, 0.500), < 0.001, 0.007
Others	22/23 (95.65%)	1.396 (0.179, 10.864), 0.750, 0.755	1.106 (0.136, 8.994), 0.925, 0.925	21/23 (91.30%)	0.435 (0.093, 2.040), 0.291, 0.655	0.257 (0.051, 1.285), 0.098, 0.229

^a^Adjusted for gender, age, residence, education level, and kinship to the high-risk patient. CPR, cardiopulmonary resuscitation. OR, odds ratio. CI, confidential interval. FDR, false discovery rate.

### CPR competency-related information

53 (9.27%) had once witnessed the scene of CA, and 7 (1.22%) had ever performed CPR on an OHCA patient. 67 (11.71%) had ever been trained (mean number of training sessions: 0.26 ± 0.93). Among those trained, the mean time since last session was 21.74 ± 59.41 months. 201 (35.14%) reported AED recognition. The mean score of the 20-item knowledge test was 7.55 ± 4.50 points (Table 7).

**Table 7:** Participants’ CPR competency-related information.

Items	*N* = 572
Once witness of cardiac arrest, *n* (%)	53 (9.27%)
Once performing CPR for a human, *n* (%)	7 (1.22%)
CPR training experience, *n* (%)	67 (11.71%)
Time interval since last training, (months, mean ± SD)	21.74 ± 59.41
CPR training times, (times, mean ± SD)	0.26 ± 0.93
Recognition of automated AED, *n* (%)	201 (35.14%)
CPR knowledge score, (points, mean ± SD)	7.55 ± 4.50

CPR, cardiopulmonary resuscitation. AED, automated external defibrillator. SD, standard deviation.

Subgroup analysis of CPR knowledge test score revealed that deficits were more pronounced among rural residents, those with primary education or below than their counterparts (rural *vs.* urban: adjusted *β* = 1.578, 95% CI = 0.762–2.394) (primary or below *vs.* secondary: adjusted β = 1.413, 95% CI = 0.255–2.571; primary or below *vs.* college or above:adjusted *β* = 4.072, 95% CI = 2.626–5.518) after adjusted for factors of gender, age, residence, education level, and kinship (Table 8).

**Table 8:** Comparisons of CPR knowledge score across subgroups by univariate and multivariate models adjusted for gender, age, residence, education level, and kinship.

Items	CPR knowledge score, (points, mean ± SD)	Crude β (95%CI), *p*, *P*_FDR_	Adjusted β (95%CI)^a^, *p*, *P*_FDR_
Gender			
Male	7.37 ± 4.59	Ref	Ref
Female	7.66 ± 4.45	0.295 (−0.466, 1.056), 0.447, 0.522	0.354 (−0.360, 1.069), 0.331, 0.517
Age			
< 50	8.52 ± 4.34	Ref	Ref
≥ 50	6.82 ± 4.49	−1.699 (−2.433, −0.964), < 0.001, < 0.001	−0.133 (−1.010, 0.744), 0.765, 0.765
Residence			
Rural areas	5.70 ± 4.52	Ref	Ref
Urban areas	8.35 ± 4.25	2.653 (1.879, 3.427), < 0.001, < 0.001	1.578 (0.762, 2.394), < 0.001, 0.004
Education level			
Primary or below	5.03 ± 4.45	Ref	Ref
Secondary	7.09 ± 4.15	2.061 (0.947, 3.175), < 0.001, < 0.001	1.413 (0.255, 2.571), 0.017, 0.040
College or above	10.25 ± 4.35	5.217 (3.947, 6.487), < 0.001, < 0.001	4.072 (2.626, 5.518), < 0.001, 0.004
Kinship			
Spouse	7.20 ± 4.42	Ref	Ref
Offspring	8.20 ± 4.63	0.996 (0.212, 1.780), 0.013, 0.018	0.330 (−0.514, 1.173), 0.443, 0.517
Others	7.26 ± 4.19	0.059 (−1.837,1.955), 0.951, 0.951	−0.794 (−2.613, 1.024), 0.391, 0.517

^a^Adjusted for gender, age, residence, education level, and kinship to the high-risk patient. CPR, cardiopulmonary resuscitation. SD, standard deviation. CI, confidential interval. FDR, false discovery rate.

We then examined the interaction between education and residence. The interaction term was not statistically significant (*P* for interaction = 0.620), suggesting that education and residence independently, rather than synergistically, influenced the CPR knowledge test score.

## Discussion

### The need for a framework-based study on the low bystander CPR rates

Bystander-initiated CPR rates remain critically low in China. Studies over the past three years have reported rates ranging from 4.8 to 20.3%, which are lower than those reported in some Asian developing countries such as Vietnam (22.1%) and Thailand (23.7%), and far behind the rates of 39.6–58.5% observed in developed countries (1–5, 29–32). This may be a key reason for the much lower survival-to-discharge rates in China (0.35% ~ 1.2%) compared with the global average of 8.8% (1, 29, 30, 33, 34).

Numerous studies, including those conducted in China, have explored the underlying causes of low bystander CPR rates. The identified causes can be summarized as follows: immature skills, legal concerns, fear of contagious diseases, emotional stress, lack of confidence, fear of causing injury, situational concerns, moral obligation, external rewards, recognition of intrinsic self-worth, *etc.* (6–20). Other factors, such as gender and racial bias and neighborhood socioeconomic status, also have an impact (9, 35–37). However, in our view, the conclusions of each study only examine individual factors, lacking a comprehensive and holistic analysis. This leads to an inability to determine which factors are more important and which are less important, leaving us confused and unable to decide which issues should be addressed first and which second. Several frameworks—such as “intention-focused model,” “structural equation model” and “intention-behavior model”—have been proposed by scholars to explain the logic underlying CPR awareness, decision-making, and execution (21–24). They revealed that initiating CPR is a complex, stepwise process involving perceived norms, self-efficacy considerations, environmental constraints and many others. We agree with these frameworks and argue that a comprehensive and holistic analysis is needed to distinguish primary barriers from secondary ones, thereby enabling targeted interventions to improve bystander CPR rates.

Building on the existing frameworks, we simplified the process of initiating CPR into three key steps: recognizing the need to perform CPR, deciding to perform CPR, and acting to perform CPR. The first step reflects bystanders’ general recognition of OHCA and the need for CPR. The decision step is the most complex. In the interval between becoming aware of the need to act and making a decision either to perform or refrain from CPR, bystanders experience internal conflict. They may feel motivated to step forward and help, yet simultaneously face internal concerns that hinder their action. The final step determines how bystanders actually initiate CPR and reflects their practical competence. We conducted the investigation based on this simplified framework and designed a questionnaire that allowed us to comprehensively and holistically analyze all the issues mentioned above. Notably, the “three-step” framework is only a simple explanation model and not a confirmed scientific result.

### Three key issues as leading obstacles to bystander CPR

The results revealed that, at the awareness stage, 73.95% of participants could recognize the need to perform CPR, although certain populations lagged behind. This finding suggests that years of public education efforts have contributed to a general recognition of CPR among the public, at least in terms of knowing when CPR should be performed. However, specific populations—including individuals aged ≥ 50 years, rural residents, those with lower educational attainment, and spouses (who tend to be older, similar to their patient partners)—may still face limitations due to restricted access to CPR promotion and training, as well as learning constraints (38, 39).

Regarding willingness, most participants reported strong willingness to perform CPR for their relatives and moderate-to-strong willingness for strangers, suggesting that willingness itself may not be a critical issue. Participants showed significantly stronger willingness to act for relatives than for strangers—a finding consistent with common expectations and previous studies (40, 41). Although willingness to perform CPR for strangers was lower than that for relatives, we do not think it is a critical obstacle limits their action for a stranger. Saving a life is both socially endorsed and intrinsically desired, in China as in other countries. This result aligns with previous reports that public willingness to perform CPR is not low (16, 17, 42). Nevertheless, it remains worthwhile to strive to raise willingness for strangers to the same level as that for relatives.

When examining internal concerns, these appeared to be the major barrier hindering action, with over 92% of participants reporting such concerns. Further investigation revealed that lack of confidence in skills was the most frequently cited reason—a factor that has been underestimated or subsumed by other causes in some studies (43, 44). Notably, the difference in lack of confidence between helping relatives versus strangers was not statistically significant, further indicating that this is the leading and widespread barrier. Additionally, other concerns—such as psychological fear, poor physical condition, medical litigation, and sanitary issues—were notably more pronounced when the victim was a stranger. This finding is consistent with common sense and previous studies (45, 46). In the subgroup analysis, spouses exhibited significantly more concerns than offspring, and this pattern persisted even after adjusting for demographic factors. This suggests that spouses’ psychological burden is largely unaffected by those demographic covariates. We speculate that this finding may be attributable to two reasons. First, in most cases, the emotional attachment and dependence between spouses are deeper than those between patients and their children, as children often have established their own families, which may weaken their emotional with the patients. Second, spouses typically spend more time accompanying the patient and prolonged exposure to the patient’s chronic illness may progressively erode their confidence and increase their psychological distress. This speculation can also explain their internal concerns toward strangers.

Analysis of the CPR competency-related information revealed unsatisfactory results. Only 11.71% had ever been trained, mean number of training sessions was merely 0.26 ± 0.93, and mean time since last session was 21.74 ± 59.41 months. These factors may explain the low scores on the 20-item knowledge test and the reported lack of confidence in CPR performance. Analysis of CPR knowledge score revealed that participants such as rural residents and those with primary education or below were particularly vulnerable.

In summary, although public awareness and willingness to perform CPR remain sub-optimal, the highest priority is addressing the public’s lack of confidence in their skills. Our investigation into CPR competency-related information revealed that the overall training rate and CPR knowledge score were both markedly low, which likely accounts for the widespread lack of confidence in skills. This may be attributed to several contextual factors: China remains a developing country; the survey was conducted in Shaanxi Province, an economically underdeveloped region in northwestern China; and the study population was predominantly middle-aged and older adults. These findings indicate that improving bystanders’ confidence should begin with enhancing the public’s actual competence. Second, greater attention should be paid to bystanders’ heightened concerns regarding strangers, particularly legal liability, psychological fear, and sanitary issues. This may contribute to the lower CPR rates observed for strangers compared with relatives. To address this issue, efforts should be made in incorporating educational components targeting these apprehensions, such as emphasizing that failure to perform CPR may carry greater legal liability than misconduct in CPR, that the net benefit of performing CPR outweighs that of withholding it, and recommending hands-only CPR to mitigate psychological discomfort and concerns regarding infectious disease transmission (47–51). Third, certain vulnerable subgroups warrant special attention, including individuals aged ≥ 50 years, those with lower educational levels, and rural residents, as they exhibited poorer performance across all assessed domains in our study. This vulnerability appears to stem from inequitable access to CPR training, as well as diminished learning capacity and sense of social value. Therefore, training resources should be preferentially directed toward these populations, with personalized training protocols tailored to their specific characteristics.

### Policy and practice implications

The findings above compel us to further our efforts in promoting bystander CPR execution. However, this is a considerable challenge. Bystander CPR rate is closely correlated with socioeconomic development (52). China has a vast territory with a huge population, accompanied by marked disparities in socioeconomic status and medical resources across regions and between urban and rural areas (53). The general public’s basic medical knowledge is limited, and attitudes toward mastering and performing CPR vary among individuals. Moreover, CPR skills and knowledge decline over time, necessitating repetitive training. Consequently, it is exceedingly difficult for China to ensure that every individual fully masters these competencies.

In recent decades, China has attempted to integrate CPR training into compulsory education to achieve universal promotion of CPR. However, as noted above, this approach only equips the public with basic CPR knowledge. Without in-depth understanding and practice, individuals’ confidence remains insufficient.

Recently, we proposed a charge nurse/doctor-led, family-caregiver-focused in-hospital training program. This program has the following characteristics: (a) it targets family caregivers of patients at high risk of OHCA; (b) it is led by a charge nurse or doctor; (c) it is conducted during the hospitalization period after the patient is diagnosed. We proposed this program for several reasons: (a) 80% ~ 85.8% OHCA events occur at home, and family members are typically the first responders (4, 30, 32); (b) family members have a stronger sense of responsibility, greater willingness to act, and fewer concerns about psychological discomfort, legal liability, and sanitary issues; (c) at the time of diagnosing high-risk OHCA, patients and their families are more attentive and compliant with instructions—a motivation that may diminish after discharge; (d) the hospital ward and the hospitalization period facilitate family members’ engagement in the training; (e) the involvement of a charge nurse or doctor increases the likelihood of successful implementation, since the families trust them; (f) from a national or societal perspective, this approach is more targeted and resource-efficient. As for how to implement this program specifically, we believe it varies according to the conditions of each institution. For our part, we propose integrating virtual reality environments into training. This will enhance trainees’ sense of immersion training effectiveness, and may also help alleviate the psychological fear bystanders often experience in the OHCA incidence. Regarding time commitment, we recommend a minimum of 30 min per session, with at least three training sessions. Training can be conducted for family members when patients return to the hospital for follow-up visits after discharge. Using a mobile application as an auxiliary tool to regularly deliver CPR knowledge and skills may help improve the retention of CPR proficiency.

This program is a resource-redistribution and cost-effective project. It concentrate efforts on a highly motivated population most likely to encounter OHCA at home, and utilizes existing hospital personnel and infrastructure. In fact, focusing on family members was proposed long ago, but it has not been widely promoted (54, 55). We believe this is because previous proposals did not specify how to carry it out concretely. By incorporating the elements of charge nurse/doctor leadership and in-hospital setting, our program becomes easy to implement. We do not deny the value of previous widespread public education and training, and we are not trying to increase CPR rates solely for relatives. We believe that starting with this program may more rapidly enhance the public’s overall CPR awareness and competence, and thereby also increase the bystander CPR rate for strangers. We are currently evaluating the efficacy of this approach, and results are pending. If its efficacy is confirmed, we think governments could support from aspects of government-led publicity, dedicated funding, legal liability protection, and mandatory requirements for relevant institutions to embed this program into their routine procedures.

### Limitations of this study and future directions

We believe the most important limitation of this study is that it was conducted among relatives of patients at high risk for OHCA, rather than actual bystanders at the scene of an OHCA event. Because no OHCA occurred during the study period, the participants’ reports were based on hypothetical scenarios rather than real-life events, which reduces the persuasiveness of the findings. Furthermore, among all the factors that may limit CPR performance, it is difficult to determine which plays a dominant role, as we cannot accurately measure the intensity of each factor, nor are these intensities comparable across different factors. Therefore, we believe that investigating individuals who were actually present at an OHCA scene would be more meaningful and convincing. A more rigorous approach would be to retrospectively analyze bystanders’ thoughts and actions that influenced their decision to perform or not perform CPR during OHCA events.

Other limitations include the following: (a) the study was conducted in two cities in northwestern China. Given China’s unbalanced regional development, this limits the generalizability of the findings to other regions; (b) the study recruited only family caregivers of high-risk, whose attitude and capacity of CPR may differ from those without a high-risk family member. Therefore, our findings should be generalized to the general public with caution; (c) Spouses constituted 61.54% of participants, while offspring were 34.44%. However, this distribution reflects the real-world pattern of inpatient caregiving in China rather than an enrollment bias; and (d) self-reported data on willingness and perceived barriers to action are subject to social desirability bias. Scores on CPR knowledge tests do not necessarily reflect true competence. To date, no testing method—even though manikin-based assessments may yield higher accuracy—has been validated against real-world performance through empirical research. Although the questionnaire survey used in this study cannot fully represent actual information, our findings may still provide a useful reference for designing more rigorous investigations in the future.

## Conclusion

This survey identified three priority issues contributing to the low rates of bystander-initiated CPR. First, lack of confidence in skills—largely attributable to poor actual competency—is the most frequently reported barrier to action. Second, when the victim is a stranger, bystanders exhibit significantly greater concerns regarding psychological fear, physical limitations, medical litigation, and sanitary issues. Third, vulnerable populations, including individuals aged ≥ 50 years, those with lower educational attainment, and rural residents, demonstrated poorer performance across all assessed domains. These findings suggest that improving bystander CPR rates requires a shift from broad awareness campaigns to targeted interventions that enhance actual competency, address specific concerns about acting for strangers, and prioritize training resources for disadvantaged subgroups. A charge nurse/doctor-led, family caregiver-focused in-hospital training program warrants further evaluation as a potentially effective strategy to achieve these goals.

## Data Availability

The raw data supporting the conclusions of this article will be made available by the authors, without undue reservation.
