# Supplementary material for: What limits a bystander’s action to perform cardiopulmonary resuscitation in China? Insights from a cross-sectional study conducted in Shaanxi Province
Source: Front Public Health. 2026 Aug 27;14:1902076. doi: 10.3389/fpubh.2026.1902076 (PMC13558223; doi:10.3389/fpubh.2026.1902076)
Supplement: Supplementary file 1 [file Table_1.docx]

**Supplementary material**

**Ability to recognize the need for cardiopulmonary resuscitation (CPR)**

Question 1: Have you ever heard of CPR? Correct answer:A

A. Yes ( )	B. No ( )

Question 2: If a person suddenly collapses, loses consciousness, and only has irregular intermittent gasping without normal breathing, do you think this person needs immediate CPR? Correct answer:A

A. Definitely need CPR ( )	B. Maybe need CPR ( )	C. No need, just wait for recovery ( )	D. Unsure ( )

**Validated 20-item quick knowledge test**

Question 1: What is the appropriate timing for performing CPR? Correct answer:C

A. Unknown ( )	B. Faint ( )	C. Cardiac arrest ( )

Question 2: How to assess cardiac arrest? Correct answer:D

A. Unknown ( )	B. The person cannot be awakened ( )	C. No heartbeat can be heard ( )	D. Carotid pulse cannot be felt ( )

Question 3: How to assess breath absence? Correct answer:D

A. Observe for chest rise ( )	B. Listen for exhaled breath sounds ( )	C. Feel for airflow on the face ( )	D. All of the above ( )

Question 4: Which is the first step among the three key procedures: airway open, breath and compression? Correct answer:D

A. Unknown ( )	B. Airway open first ( )	C. Breath first ( )	D. Compression first ( )

Question 5: Hard or soft surface to place the casualty before performing CPR? Correct answer:B

A. Unknown ( )	B. Hard ( )	C. Soft ( )	D. Not critical ( )

Question 6: Where is the location for chest compression? Correct answer:D

A. Unknown ( )	B. Everywhere on the chest ( )	C. In front of the heart ( )	D. The intersection of the internipple line and the midline of the sternum ( )

Question 7: Should arms remain straight or be bent during chest compression? Correct answer:B

A. Unknown ( )	B. Straight ( )	C. Bent ( )	D. Not critical ( )

Question 8: Should arms be perpendicular to the chest during chest compression? Correct answer:B

A. Unknown ( )	B. Perpendicular to the chest ( )	C. At an angle to the chest wall ( )	D. Not critical ( )

Question 9: How should the hands be positioned during chest compression? Correct answer:C

A. Unknown ( )	B. Place one hand on top of the other ( )	C. With fingers clasped around the back of the lower hand ( )	D. Not critical ( )

Question 10: What is the appropriate depth for chest compression? Correct answer:D

A. Unknown ( )	B. Less than 3cm ( )	C. 3~5cm ( )	D. more than 5cm ( )

Question 11: What is the rate for chest compression? Correct answer:C

A. Unknown ( )	B. Less than 100 per min ( )	C. 100~120 per min ( )	D. more than 120 per min ( )

Question 12: Should hands lift off the chest during chest compression? Correct answer:C

A. Unknown ( )	B. Necessary ( )	C. Unnecessary ( )	D. Not critical ( )

Question 13:How many chest compressions should be performed before opening the airway and delivering rescue breaths Correct answer:B

A. Unknown ( )	B. 30 ( )	C. 60 ( )	D. 100 ( )

Question 14: Is it necessary to clear airway obstructions from the mouth and nose? Correct answer:B

A. Unknown ( )	B. Necessary ( )	C. Unnecessary ( )	D. Not critical ( )

Question 15: Should the nose be pinched during rescue breaths? Correct answer:B

A. Unknown ( )	B. Necessary ( )	C. Unnecessary ( )	D. Not critical ( )

Question 16: Is it necessary to tilt the head and lift the chin during rescue breaths? Correct answer:B

A. Unknown ( )	B. Necessary ( )	C. Unnecessary ( )	D. Not critical ( )

Question 17: What is the duration for rescue breaths during CPR? Correct answer:B

A. Unknown ( )	B. More than 1 second ( )	C. More than 2 seconds ( )	D. More than 3 seconds ( )

Question 18: How many rescue breaths should be given per cycle during CPR? Correct answer:C

A. Unknown ( )	B. 1 ( )	C. 2 ( )	D. 3 ( )

Question 19: What is the compression-to-ventilation ratio during CPR? Correct answer:B

A. Unknown ( )	B. 30:2 ( )	C. 50:2 ( )	D. 60:2 ( )

Question 20: After how many cycles of CPR should vital signs be reassessed? Correct answer:C

A. Unknown ( )	B. 3 ( )	C. 5 ( )	D. 10 ( )
